# Cemented Hemiarthroplasty in Elderly Osteoporotic Unstable Trochanteric Fractures using Fracture Window

**DOI:** 10.5704/MOJ.1603.002

**Published:** 2016-03

**Authors:** A Thakur, M Lal

**Affiliations:** Department of Orthopaedics, Indira Gandhi Medical College, Shimla, India

**Keywords:** Unstable, Osteoporotic, Intertrochanteric, hemiarthroplasty, cemented

## Abstract

**Introduction:** We would like to analyze the role of cemented hemiarthroplasty in elderly osteoporotic unstable intertrochanteric fractures through trochanteric fracture window.

**Materials and Methods:** The study was conducted from July 2011 to July 2014. From a total of 265 consecutive patients with intertrochanteric fractures of 42 patients were selected according to inclusion criteria and results were analyzed prospectively. All patients were operated at tertiary care institute. Patients which matched the inclusion criteria were selected. 42 patients entered the study and all completed the study. Primary cemented hemiarthroplasty was done in all patients. Modified Harris Hip Score was used to assess all the patients.

**Results:** 42 patients were included in the study with an average age of 80.7 years. Only AO/OTA type 31-A2.2 and 31-A2.3 were included, average HHS at final follow up of three years was 86.2. No revision or reoperation was done.

**Conclusion:** In a selected cohort of patients primary prosthetic replacement in elderly osteoporotic unstable intertrochanteric fractures is good option and the surgical technique allowed us to perform it more easily.

## Introduction

Intertrochanteric fractures are usually seen in adults with a fair number seen in elderly individuals. Osteoporotic hip fracture is an established health problem in the West and is increasingly recognized as a growing problem in Asia. With rising life expectancy throughout the globe, the number of elderly individuals is increasing in every geographical region, and it is estimated that the incidence of hip fracture will rise from 1.66 million in 1990 to 6.26 million by 2050^1,2^.

The management of these fractures evolved from conservative approach, with the help of skeletal traction to operative procedures such as fixed angle blade plates, sliding hip screw and lately the intramedullary devices. In fractures with inherently stable configuration the results of osteosynthesis were good but the review of literature reveals that the results of osteosynthesis in unstable intertrochanteric fractures were poor.^3^

These implants had their success when bone quality is good such as when the fracture occurs in otherwise healthy adult, but in elderly individuals with osteoporotic bone the complication rate is high such as screw cut out from head, excessive collapse of fragments leading to shortening, implant breakage and pull out. Though considerably less with intramedullary implants but screw back out and implant breakage still remain, when early return to activity was aimed for, as was deemed necessary in case of elderly individuals^4^. Prosthetic replacement of the femoral head, with a great success in femoral neck fractures, appears to be a better alternative in unstable intertrochanteric fractures as it would provide rapid and early rehabilitation which is necessary in elderly individuals to reduce morbidity and mortality^5,6^. The purpose of our study is to analyze the role of hip arthroplasty in cases of unstable osteoporotic intertrochanteric femur fractures.

## Materials and Methods

The study was conducted from July 2011 to July 2014 at our institute and results were analyzed prospectively. Institutional Review Board clearance was taken before the start of study. From a total of 265 consecutive cases of intertrochanteric fractures 42 cases were selected which fulfilled the inclusion criteria. Informed consent was taken from each patient. The inclusion criteria were age more than 60 years with no upper limit, confirmed osteoporosis (on DEXA Scan Bone-Mineral Density T-Score greater than -2.5), an unstable fracture configuration limited to AO/OTA 31-A2.2 and 31-A2.3 only. The study involved a total of 42 cases followed for a maximum period of 3 years and minimum of one year. There were 40 females and only 2 males. Average duration from injury to surgery was 3 days excluding two patients who had presented late i.e. 46 days and 2 months after injury. Polytrauma patients, pre-existing hip infections, pathological fractures and fractures with stable configuration were excluded from study.

### Surgical Technique

All patients were operated in lateral position using standard posterolateral approach. After skin incision, Tensor Fascia Lata was cut and fibers of gluteus maximus were separated along the line of incision. Fascial incision was extended distally far enough to expose the tendinous insertion of the gluteus maximus on the posterior femur. Thereafter fracture geometry was assessed and fracture lines palpated. We deviated from the standard posterolateral approach from here. In 15 cases fracture line was directly visible, while in 27 cases intact sleeve of gluteus medius and vastus lateralis was covering it. Interval was developed along the facture line directly (n=15) and in cases where it was only palpated (n=27) the interval was developed with the help of electric cautery, by palpating along the fracture line. The interval was developed in coronal or oblique plane and followed proximally into Gluteus Medius (Fig 1a). The two parts of Gluteus medius (anterior and posterior) along with the bony attachment meaning the fracture fragments of greater trochanter were retracted to expose the fractured neck and head (Fig 1b). The head along with the part of the fractured neck femur was extracted with the help of bone holding forceps (Fig 1c). The Acetabulum is cleared of pulvinar and trial cup was inserted. The femoral preparation was done with manufacturer supplied reamers and broaches.

**Fig. 1a:**
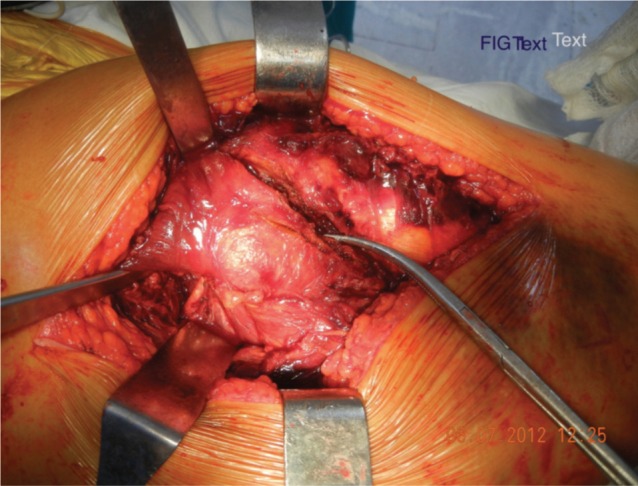
Identification of fracture Line.

**Fig. 1b:**
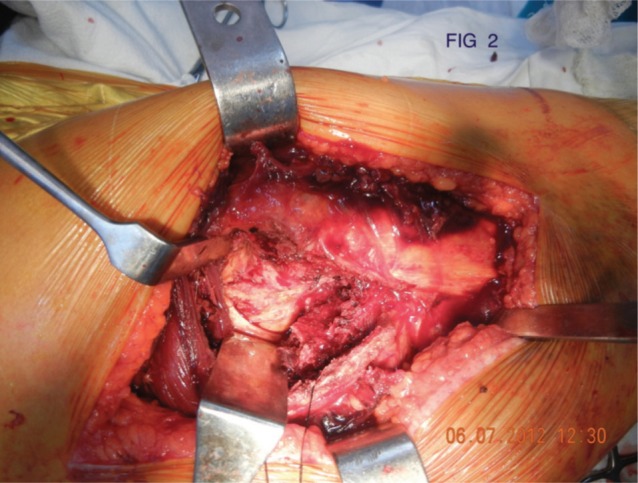
Retraction of Fracture Fragments for creating fracture window.

**Fig. 1c:**
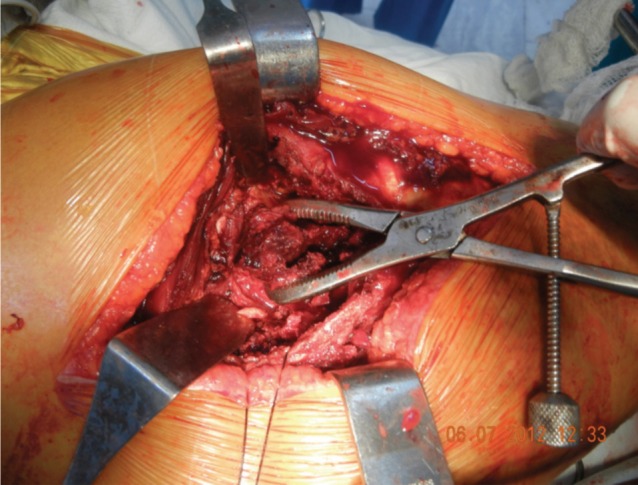
Extraction of Femoral Head and Neck through fracture window.

After femoral preparation three drill holes were made over the lateral aspect of the proximal femoral shaft about 4 to 5 centimeters distal to the fracture site, with about one centimeter interval between the holes. Two stainless steel cerclage wires were passed through these holes outside to inside exiting through the consecutive hole (Fig 1d). Now the four free ends were lying on the lateral aspect of the proximal femur. The trial stem was inserted taking care of the anteversion, which was judged by the long axis of the leg. The trial stem was sunk adequately to achieve equal limb length, which was also checked by the Shuck test with displacement of not more than 2-3 millimeters. The trial reduction was performed and stability of hip was assessed. Before the final implant was inserted, cementing was done using second generation cementing technique.

**Fig. 1d:**
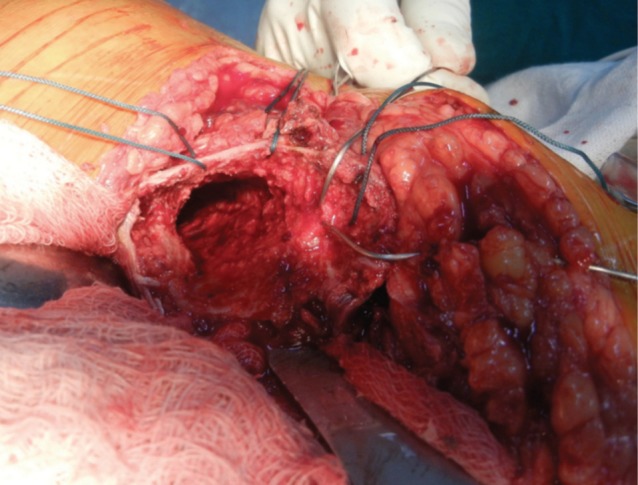
Passing of Wire Loops and Ethibond.

We reconstructed the medial calcar with the help of cement mantle in all the cases (Fig 1e). After reduction of the hip with the final implant, the main fracture fragments of the greater trochanter were approximated to each other with help of non-absorbable ethibond sutures (Fig 1f).

**Fig. 1e:**
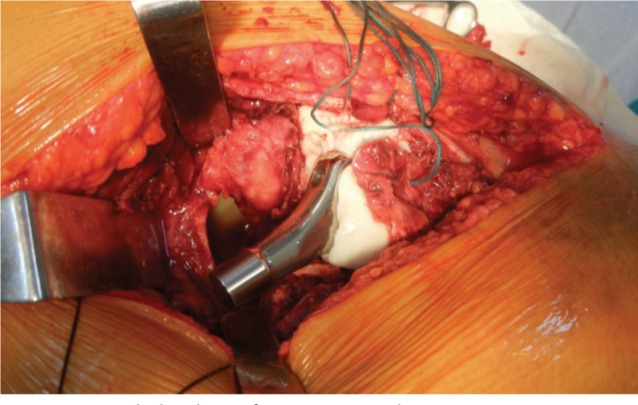
Medial Calcar of cement Mantle.

**Fig. 1f:**
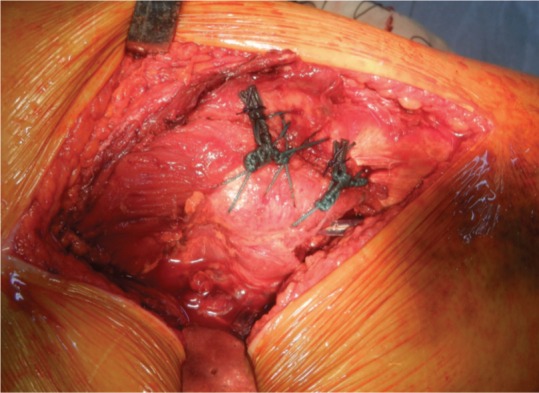
Closure of fracture window.

Then two cerclage wires were passed just superior to greater trochanter within the insertion of gluteus medius, inside out which comes to lies over the substance of the muscle. These cerclage wires were then tied to the cerclage wires previously attached to proximal femur and adequately tightened. Rest of the soft tissue closed in layers over negative suction drains. We used single shot third generation cepahalosporins pre-operatively, intra-operatively at the time of induction and continued post-operatively for 2 days. Primary wound inspection was done on 2nd post-operative day and all drains were removed. Sideways sitting started after check x-rays on following day. Patient was allowed walking with the help of walker after drain removal. Initially partial weight bearing was stared for 2-3 days and then full weight bearing as tolerated by the patient. Patients were discharged on 14th post-operative day and followed at 6 weeks, 12 weeks, 24 weeks, one year, two year and three year. Patients were assessed on the basis of their self-assessment regarding physical, social, mental well being and relief in pain. Modified Harris hip score was used to standardized the results. Radiographs were done at each visit for radiological assessment of stem sinking, aseptic loosening, malpositioning of stem.

## Results

The study was conducted during July 2011 to July 2014 at our institute and was analyzed prospectively. Study involved a total of 42 cases followed for a maximum period of 3 years and minimum of one year. There were 40 females and only 2 males. Average age in our study was 80.7 years with range from 65 to 96 years. Average duration from injury to surgery was 3 days, excluding two patients who had presented late meaning 46 days and 2 months after injury. The fractures were classified according to AO/OTA classifications and only AO/OTA type 31-A2.2 and 31-A2.3 were included.

All patients were ambulatory before injury independently (n=27) or with help of cane (n=15). All patients were thoroughly investigated for any comorbid conditions such as hypertension (n=23), diabetes (n=13), and combination of both (n=5) and one patient had Alzheimer’s disease.

The average duration of surgery was 96 min (range 70-112), with a mean blood loss of 125 ml (range 100-250 ml); all patients were given postoperative blood transfusions, considering the elderly state of the patients. All patients were out of the bed and mobile with help of walker with average of 2.6 days (range 2-4 days). The average duration of hospital stay was 17.5 days as we routinely discharged the patients after suture removal on 14th postoperative day.

We encountered three complications, of which one was superficial infection, second was urinary tract infection and third was superficial bed sore. There was no dislocation, aseptic stem loosening, osteolysis or subsidence of stem. We did encounter late complication in the form of wire breakage in 6 cases. There was no significant limb length discrepancy with an average of less than one centimeter.

All patients were assessed according to modified Harris hip score at the time of discharge, 6 weeks, 12 weeks, 6 months, one year, two year and three year follow up. Two patients were graded excellent, 17 were good, 22 were fair and one was poor at 3 months follow up. At final follow up of mean duration of 16.5 months (12 – 36 months) 19 patients were graded as excellent, 17 were good and 6 were fair. Average HHS improved from 74.4 (range 64-91) at three months follow up to 86.2 (range 74-94) at final follow up. All patients were ambulatory at final follow up, 25 with cane and 17 without any support. We assessed for abductor lurch gait and found it in 35 patients at three months follow up but it improved and only three patients had lurch at final follow up. Patient’s demographic data is shown in Table 1. Radiographs of the cases are shown in Fig 2a-2c.

**Table I:** Demographic detail of 42 patients

Patients	Age (Years)	Sex	Fracture Type	Bone Mineral Density Level (Dexa Scan Values: T- Score)	Fixation Type	Modified Harris Hip Score at Final Follow Up	Limb Length Difference (cm) at Final Follow-up
1	84	Female	31–A2.3	2.6	Steel Wires	88	1
2	76	Female	31–A2.2	2.5	Ethibond Sutures	90	1.5
3	65	Female	31–A2.2	2.5	Ethibond Sutures	92	1
4	83	Female	31–A2.3	2.6	Steel Wires	90	1.5
5	94	Female	31–A2.3	3.1	Steel Wires	76	1.5
6	78	Female	31–A2.2	2.5	Ethibond Sutures	93	1
7	90	Female	31–A2.3	3.0	Steel Wires	77	1.5
8	88	Female	31–A2.3	2.8	Steel Wires	84	1.5
9	79	Female	31–A2.2	2.5	Ethibond Sutures	90	1.5
10	68	Female	31–A2.2	2.5	Steel Wires	94	1
11	73	Female	31–A2.2	2.6	Ethibond Sutures	92	Less Than 1
12	77	Female	31–A2.2	2.6	Steel Wires	93	Less Than 1
13	84	Female	31–A2.2	2.6	Steel Wires	80	1
14	81	Female	31–A2.2	2.7	Ethibond Sutures	82	1
15	88	Female	31–A2.3	2.9	Steel Wires	82	Less Than 1
16	96	Female	31–A2.3	3.3	Steel Wires	74	1
17	77	Female	31–A2.2	2.6	Steel Wires	92	Less Than 1
18	87	Female	31–A2.3	3.0	Steel Wires	80	Less Than 1
19	76	Female	31–A2.2	2.8	Ethibond Sutures	93	1
20	74	Female	31–A2.2	2.8	Steel Wires	94	Less Than 1
21	82	Female	31–A2.2	2.6	Steel Wires	86	Less Than 1
22	87	Male	31–A2.3	2.6	Steel Wires	80	Less Than 1
23	92	Female	31–A2.3	3.0	Steel Wires	74	1
24	85	Female	31–A2.3	2.9	Ethibond Sutures	80	1.5
25	83	Female	31–A2.2	2.6	Steel Wires	82	1.5
26	89	Female	31–A2.3	2.9	Steel Wires	80	1.5
27	75	Female	31–A2.2	2.7	Steel Wires	92	1.5
28	89	Female	31–A2.3	3.0	Steel Wires	76	1
29	90	Female	31–A2.3	3.0	Steel Wires	78	1.5
30	81	Female	31–A2.2	2.8	Ethibond Sutures	88	1.5
31	80	Male	31–A2.2	2.5	Ethibond Sutures	88	Less Than 1
32	72	Female	31–A2.2	2.5	Ethibond Sutures	93	Less Than 1
33	77	Female	31–A2.2	2.7	Steel Wires	92	1
34	71	Female	31–A2.2	2.5	Steel Wires	94	Less Than 1
35	87	Female	31–A2.3	2.6	Steel Wires	80	1.5
36	81	Female	31–A2.2	2.7	Steel Wires	90	1
37	84	Female	31–A2.2	2.7	Steel Wires	80	1.5
38	72	Female	31–A2.2	2.5	Steel Wires	92	Less Than 1
39	69	Female	31–A2.2	2.5	Steel Wires	94	Less Than 1
40	82	Female	31–A2.2	2.6	Steel Wires	84	1.5
41	79	Female	31–A2.2	2.5	Steel Wires	88	1
42	68	Female	31–A2.2	2.5	Steel Wires	94	Less Than 1

**Fig. 2a:**
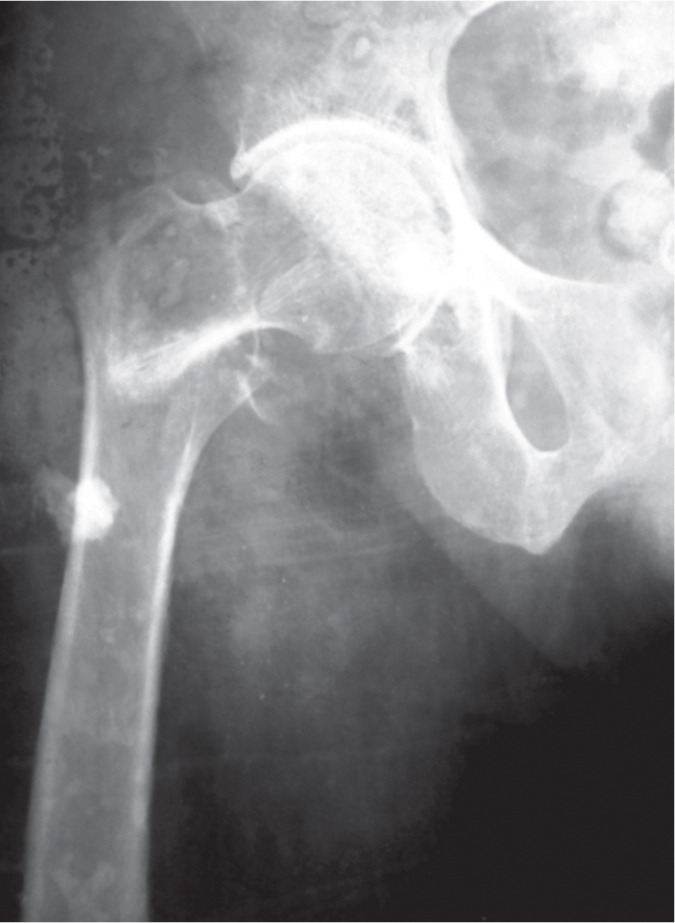
Preoperative Radiograph AP.

**Fig. 2b:**
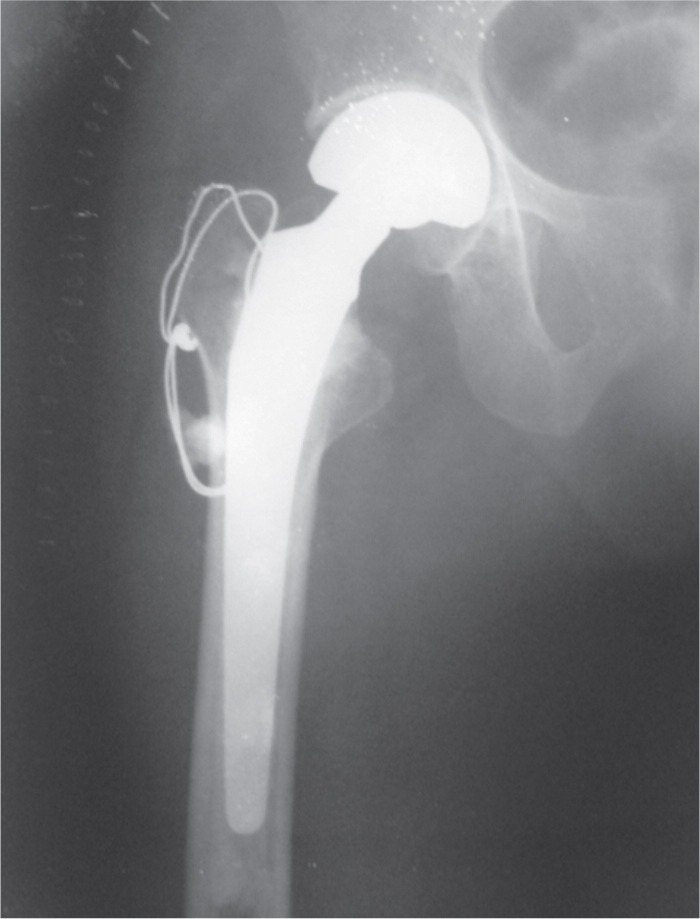
Postoperative Radiograph AP view.

## Discussion

Management of unstable intertrochanteric fractures especially in osteoporotic patients remains controversial^7^. Internal fixation had its share of success in stable fractures with good bone stock, but all types of fixation devices such as dynamic hip screw, angle blade plate and lately the intramedullary devices have failed to demonstrate the same success rate in complex unstable and osteoporotic intertrochanteric fractures^8^. Various complications such as screw cut out, screw back out, plate pull out, varus collapse of fracture, rotational deformity and limb shortening occur with these fixation methods. Mal-union and non-union are common problems in follow-up periods in patients treated with internal fixation devices. Patients presenting again with these problems presents an entirely different picture and management then is very difficult. Early rehabilitation with internal fixation devices used in unstable osteoporotic fractures is fraught with complications^9^. Overall expenses increases as revision surgeries are done and morbidity of patient increases along with mortality^10^.

Primary prosthetic replacement in these unstable osteoporotic intertrochanteric patients is not associated with all these complications. Although a technically demanding surgery but if performed meticulously it is associated with lower complication rates. Enough evidence is present in literature to support hemiarthroplasty as a primary treatment option in such types of fractures^11-14^. As it allows early weight bearing and there is no fear of varus collapse of fracture fragment, primary prosthetic replacement is the method of choice. In patients treated with hemiarthroplasty, rehabilitation is quick and the complications such as bedsores, chest infection and atelectasis are markedly lower. It allows early return to pre-fracture activity level and essentially prevents the aggravation of comorbid conditions. Ability to bear weight in early post-operative period is the key to success of hemiarthroplasty.

Average age in our study was 80.7 years which was comparable to the other studies, thereby suggesting that we targeted the same age group. The Mean Harris hip Score at final follow up was 86.4. Most of the patients returned to their pre operative activity level, 64% patients were able to walk without support preoperative, 40% of patients were able to walk without any support at final follow up, while 36% patients of total were using cane before surgery, and 60% were dependent on cane after surgery at final follow up. Stern and Goldstein reported on 29 patients with intertrochanteric fractures treated with the Leinbach prosthesis with excellent results in 88%. They reported a deep infection rate of 6.8% but no dislocations^15^. Stern and Angerman reported on 105 cases of unstable intertrochanteric femoral fractures treated with Leinbach prosthesis. They reported a deep infection rate of 2.8%. They obtained a 94% success rate in returning the patient to the pre-fracture ambulatory status^16^. Grimsrud *et al* (2005), reported in their study of 39 patients of unstable intertrochanteric fractures treated with cemented hemiarthroplasty that cerclage wire fixation of fracture fragments is an effective method and early weight bearing can be allowed without complications^17^. We had 7% complications rate with no deep infections, which is comparable to the other studies, all of which were managed conservatively. There was no re-operation in our study.

Use of calcar replacement prosthesis is desirable but it is expensive. In all patients we recreated the calcar with the help of cement mantle. The anatomy was restored and the resultant complex provided stable configuration. There was an average of less than one centimeter of limb length discrepancy at final follow up in our study.

Total hip arthroplasty has also been used in the management of these fractures but in our opinion it increases both the magnitude and cost of surgery, although the functional outcome is good but the dislocation rate is higher as compared to hemiarthroplasty in elderly individuals.

All the approaches described for hemiarthroplasty in literature have comparable dislocation rates. Use of trans trochanteric approach had been associated with lurching gait as mentioned by Astvaldur *et al*. The fixation of greater trochanter to remaining construct with the help of stainless steel wires and non absorbable sutures had to be meticulous to avoid the lurching in the early post operative period. We had three cases of lurching gait at final follow up. There was no dislocation in our study.

In our series we have excellent and good results in about 86% of cases using the Harris hip-scoring system. In our study the complication rate is low due to meticulous surgical techniques but it is only in short-term follow-up. Long term complications such a stem failure, loosening, protusio acetabuli, late infections and dislocations were not seen as duration of maximum follow-up was only 3 years. Our study is limited by the fact that the cohort size is small and follow-up duration is less. It should also be mentioned that we have selected the cases meticulously after proper planning as we thought these were the cases which would be benefitted most by the primary prosthetic replacement. Further, a comparison was not done with internal fixation devices, which limits our study.
